# Resident-reported evaluation of Party-Mass Service Centers in China: A cross-sectional analysis of participation and governance context

**DOI:** 10.1371/journal.pone.0359412

**Published:** 2026-09-28

**Authors:** Haibo Yao, Qirui Zhang, Huaizhi Xu, LiFa Hu

**Affiliations:** 1 Yangzhou University Social Governance Innovation Research Center, School of Marxism, Yangzhou University, Yangzhou, Jiangsu, China; 2 School of Art and Design, Yangzhou University, Yangzhou, Jiangsu, China; 3 Lianyungang Technical College, Lianyungang, Jiangsu, China; Universitas Prima Indonesia, INDONESIA

## Abstract

Party-Mass Service Centers (PMSCs) combine grassroots service delivery, participation, and Party-led community governance, yet little is known about how residents evaluate these institutions. Using a mixed-mode nonprobability survey of 2,688 residents linked to 140 PMSCs in Hanjiang District, Yangzhou, this study conceptualizes resident evaluation as a reported, reference-dependent judgment rather than a direct measure of institutional performance. Evaluation was strongly ceiling-concentrated (M = 4.747, median = 5.000; 63.3% strict top box). Center-clustered OLS showed that, relative to nonparticipants, residents who participated rarely, occasionally, often, or always reported evaluation scores higher by 0.298, 0.370, 0.343, and 0.406 points, respectively (all p < .001). A factor-score model and center-grouped binomial GEE yielded consistent results; adjusted top-box probability rose from 47.8% among nonparticipants to 82.6% among always-participants. Between-center variation was small (ICC = 0.011), and sensitivity analyses did not materially alter the participation pattern. The study contributes by separating reported evaluation from objective performance, showing the robustness of the participation-evaluation association under severe ceiling concentration, and demonstrating how center-aware inference can strengthen resident-centered analysis of grassroots governance. Findings remain associational because the design is cross-sectional and nonprobability-based.

## 1. Introduction

Public-service evaluation offers a resident-centered perspective on how public institutions are encountered and assessed. Administrative indicators document what institutions provide, whereas survey evaluations capture how services, institutional arrangements, and interactions are perceived by those who experience them. Research on citizen satisfaction shows that reported evaluations reflect perceived performance as well as the expectations against which performance is judged [1,2]. Recent public-administration scholarship likewise emphasizes citizens’ lived encounters with the state, including frontline service delivery and communication [3,4]. Resident-reported evaluation is therefore analytically valuable, but it should not be treated as equivalent to objective institutional performance.

This distinction is especially relevant to Party-Mass Service Centers (PMSCs) in China. PMSCs are place-based grassroots platforms that connect residents with Party organizations, public and community services, and opportunities for community engagement. Their functions can include service information, community activities, participation and consultation channels, and routine interactions with grassroots governance institutions. Historical research on neighborhood and shequ governance documents the development of local state-society interfaces in urban China [6–8], while more recent work emphasizes the service-oriented and organizationally diverse character of grassroots governance [9,10].

The multifunctional character of PMSCs makes the meaning of resident evaluation particularly important. This study treats evaluation as a reference-dependent, experience-based, and context-conditioned judgment rather than a direct audit of institutional quality. Residents may assess similar institutional conditions against different expectations, prior bureaucratic experiences, or comparison points. Consequently, the same numerical rating need not reflect the same evaluative standard across respondents.

The empirical outcome is therefore reported survey evaluation: residents’ recorded responses to the evaluation items. These responses indicate an underlying judgment but do not exhaust it. Reported evaluations may also be sensitive to response context. Because the survey did not directly measure social desirability, the analysis neither identifies nor quantifies social-desirability bias; response context is treated only as an interpretive consideration.

This distinction matters because evaluations are strongly concentrated at the upper boundary. A continuous mean score remains interpretable, but variation among highly positive respondents is compressed. The analysis therefore uses complementary outcome representations: the mean evaluation score, a standardized factor score, and a strict top-box indicator. These alternatives do not remove the ceiling effect; they test whether the observed participation-evaluation association depends materially on how the concentrated outcome is represented.

The study retains a broader resident-side framework of participatory embeddedness, service visibility, administrative accessibility, and governance friction. Within that framework, the inferential analysis focuses on participation frequency and asks whether resident-reported evaluation differs systematically across participation categories after demographic adjustment and center-aware inference. This narrower empirical focus is consistent with the cross-sectional design and avoids treating contemporaneous associations as causal mechanisms.

The study makes three contributions. First, it distinguishes objective institutional performance, underlying evaluative judgment, and the survey evaluation actually observed. Second, it brings a resident-centered perspective to research on China’s grassroots governance by examining participation in relation to evaluation of a multifunctional local institution. Third, it addresses a severe ceiling distribution with complementary outcome specifications and explicitly accounts for clustering within service centers. These contributions are deliberately bounded: the mixed-mode nonprobability, cross-sectional design supports within-sample associations rather than causal or population-representative inference.

## 2. Literature review and analytical framework

### 2.1 Resident evaluation as a reported and reference-dependent judgment

Citizen satisfaction and public-service evaluation research has long challenged the assumption that survey ratings directly mirror service performance. Van Ryzin’s expectancy-disconfirmation model emphasizes comparison between perceived performance and expectations [1], and a recent meta-analysis confirms the central role of both performance perceptions and expectations while showing that measurement and research design shape observed results [2]. The implication is straightforward: a reported rating is meaningful, but it is a judgment formed relative to a reference standard rather than a direct administrative performance indicator.

Recent public-administration research extends this perspective by examining citizens’ lived experiences in interactions with the state. Administrative-burden research synthesizes how citizens experience public institutions, while work on public encounters emphasizes the interactional dynamics of frontline contact [3,4]. These literatures reinforce the need to distinguish institutional conditions from how those conditions are experienced and reported.

Reported evaluations may also matter beyond the immediate service encounter. Comparative evidence links satisfaction with public-service performance to trust in local government [5]. Although trust is not an outcome in the present study, this literature illustrates the wider relevance of citizen-reported evaluation without making it equivalent to independently observed performance or a fully observed latent judgment.

### 2.2 Party-Mass Service Centers and grassroots governance

Research on neighborhood governance in China provides the institutional context for PMSCs. Historical studies describe how neighborhood organizations and shequ reforms developed as grassroots interfaces connecting state administration, community organization, welfare provision, and social coordination [6–8]. These works remain foundational because they document the institutional trajectory from which contemporary service-oriented grassroots platforms emerged.

More recent research characterizes urban grassroots governance as increasingly oriented toward public service and social management while remaining diverse in organizational form [9,10]. PMSCs can therefore be understood as multifunctional local interfaces rather than narrow administrative counters. Institutional descriptions alone, however, do not reveal how residents evaluate participation, information, service access, and everyday governance interaction. This resident-side gap motivates the present analysis.

### 2.3 Participatory embeddedness, service visibility, and administrative accessibility

Participatory embeddedness captures the extent to which residents are connected to the institutional setting through participation and engagement. Public participation in China takes heterogeneous forms, and informational or consultative practices are more common than arrangements involving substantial sharing of decision authority [12]. Formal participation should therefore not be equated with substantive influence. Community-level studies likewise show that participation can be tokenistic, while informal engagement may become consequential where formal channels provide limited responsiveness [13,14]. These findings support an expectation of association, not a causal mechanism: more frequent participants may report different evaluations, but favorable evaluation may also increase willingness to participate.

Service visibility concerns whether residents know what services and information channels are available. Transparency research increasingly asks not only how much information is disclosed, but how citizens receive, interpret, and use it [11]. For PMSCs, breadth of service awareness and recognition of information channels are therefore conceptually distinct.

Administrative accessibility refers to the practical legibility of entry points into service provision. Recognizing service windows differs from general service awareness because it concerns whether residents can identify where particular forms of assistance or consultation are organized. Accessibility may be related to reported evaluation without implying that this cross-sectional study identifies a causal effect.

### 2.4 Governance friction and administrative burden

Governance friction refers to reported problematic patterns in participation or service interaction, including being registered without appearing, appearing without participating, or participating with low engagement. The measure does not reproduce the full administrative-burden construct, but that literature provides a useful interpretive lens. The foundational framework distinguishes learning, compliance, and psychological costs in citizen-state interactions [15], and recent synthesis work emphasizes how such burdens are experienced across public encounters [3].

Because friction and evaluation are contemporaneous self-reports, any covariance between them cannot establish temporal ordering, causal direction, or an independently observed institutional mechanism. Governance friction is therefore retained as part of the broader interpretive framework rather than treated as evidence of a verified causal pathway.

### 2.5 Analytical expectation and empirical scope

The four-dimensional framework organizes the resident-side context of evaluation, but the principal inferential test concerns participation frequency. The central expectation is that participation frequency is associated with resident-reported evaluation, with no assumption that participation precedes evaluation or that the association is linear across all categories.

The analysis evaluates this association across the continuous mean score, a standardized factor score, and a strict top-box outcome, while accounting for within-center dependence. Other measured dimensions – volunteering, service awareness, information channels, service-window recognition, and governance friction – remain part of the study’s measurement and interpretive context but are not presented as independently estimated focal associations in the models reported below.

## 3. Research design and methods

### 3.1 Research setting and study design

This cross-sectional study was conducted in Hanjiang District, Yangzhou, Jiangsu Province, China. To preserve contextual transparency while limiting unnecessary identification at lower administrative levels, the district is named in full, whereas subdistricts, townships, development zones, villages, communities, and individual centers are anonymized in the analytical materials.

Fieldwork was conducted from 1 June to 31 August 2025 and covered all 146 village- and community-level PMSCs in the district. The research team visited each center, observed its spatial layout, service facilities, administrative windows, and activity arrangements, and held thematic discussions with staff about organizational operation, service provision, resident participation, and grassroots governance practices. Alongside this institutional investigation, residents and villagers were surveyed about their awareness, participation, service experiences, and evaluations of the centers.

The study therefore comprised two analytically distinct components. The institutional field investigation achieved full coverage of 146 PMSCs, while the resident-level analytical dataset contained 2,688 valid questionnaires associated with 140 distinct centers. Accordingly, 146 refers to institutional field-investigation coverage and 140 to the center clusters represented in the resident analytical sample.

Resident observations were clustered within centers. Across the 140 represented centers, cluster size ranged from 1 to 83 respondents (mean = 19.2; median = 18), and 12 centers were represented by a single respondent. These unequal cluster sizes were retained rather than altered through post hoc deletion; the resident sample forms the basis of the statistical analyses below.

### 3.2 Questionnaire administration and sampling approach

The resident questionnaire used a mixed online-offline design. Offline, the research team distributed paper questionnaires during field visits to residents and villagers encountered on site or in the immediate vicinity of the service centers. Online, the survey was administered through Wenjuan (Wenjuan.com), with respondents accessing the questionnaire by QR code or survey link. The questionnaire content was identical across modes, although mode-specific measurement equivalence was not directly tested.

Recruitment occurred during the same fieldwork period, from 1 June to 31 August 2025. The mixed-mode approach broadened practical access to respondents with different levels of digital access and response convenience, but it did not constitute probability sampling. The resident survey is therefore treated as a mixed-mode nonprobability sample based on field interception and voluntary online participation [16].

This sampling structure is a design-level constraint on interpretation. Residents who were more familiar with PMSCs, more engaged in center-related activities, more willing to speak with field researchers, or more motivated to complete an online survey may have been more likely to enter the sample. Consequently, the observed distributions of participation and reported evaluation, and the magnitude of associations between them, may differ from those under probability-based recruitment.

The analyses therefore estimate adjusted within-sample associations rather than population-representative levels or causal effects. Neither covariate adjustment nor the center-aware procedures described below removes self-selection arising from the nonprobability sampling process.

### 3.3 Ethics

The study protocol was reviewed by the Scientific Research Management Office (Ethics Review Working Group) of the School of Marxism, Yangzhou University, and was determined to be exempt from formal ethics review as a low-risk anonymous social questionnaire study (No. YZU2025022103; 21 February 2025). The questionnaire collected routine non-sensitive information and did not request personally identifying information. Participation was voluntary. Written informed consent was obtained from offline participants before questionnaire completion, and electronic informed consent was obtained from online participants before access to the survey. For participants under 18 years of age, informed consent was obtained from a parent or legal guardian before participation.

### 3.4 Measures

#### 3.4.1 Resident-reported evaluation.

The primary outcome was constructed from 20 Q19 evaluation items, each recorded on a five-point scale from 1 = very inconsistent to 5 = very consistent. All 20 items had valid values and no missing responses in the analytical sample.

Measurement properties were assessed before construction of the composite outcome [17]. Internal consistency was very high (Cronbach’s α = 0.9862, 95% CI [0.985, 0.987]), and factorability was strong (KMO = 0.9838; Bartlett’s χ² = 80,067.596, p < .001). The eigenvalue pattern, scree plot, and a 500-iteration permutation-based parallel analysis all supported one dominant factor.

A one-factor MINRES model without rotation produced loadings of 0.786–0.922 and SS loadings of 15.7277, accounting for 78.64% of variance. The primary continuous outcome was the mean of the 20 items on the original 1–5 metric. A standardized one-factor score was retained as an alternative outcome and correlated 0.9995 with the mean score.

The strict top-box outcome was coded 1 only when all 20 items were rated 5 (equivalent to a Q19 mean of 5.000) and 0 otherwise. This criterion identified 1,701 respondents (63.281%). The mean score preserves the original metric, the factor score tests sensitivity to equal weighting, and the top-box indicator directly represents the mass at the upper boundary; none is assumed to eliminate the ceiling effect.

#### 3.4.2 Participation and other study measures.

Participation frequency was measured in five categories: never, rarely, occasionally, often, and always. In the inferential models, participation was treated categorically, with never as the reference group. The respective category counts were 742, 717, 614, 393, and 222.

Volunteer status was coded 1 = yes and 0 = no. Service visibility measures included a count of 19 predefined service categories known and a count of seven information channels known. Administrative accessibility was represented by the number of recognized service-window types among four predefined categories. Multiple-response variables were coded by matching formal response options rather than mechanically splitting response strings, because some official options contained Chinese enumeration punctuation.

Governance friction was a count of three reported problem types: being registered but not appearing, appearing without participating, and participating with low engagement. Each component was coded 0/1 and summed; a separate no-problem response contributed zero. The validated data contained no remaining logical conflicts between the no-problem response and the component indicators.

#### 3.4.3 Demographic covariates.

The models adjusted for gender, age, educational attainment, income category, and Party membership. Gender was coded 0 = female and 1 = male. Age was modeled categorically with 30–45 years as the reference group; education used high school or below as the reference category; income used 3,000–6,000 yuan as the reference category; and Party membership was coded 0 = no and 1 = yes. Table 1 summarizes demographic characteristics, and Table 2 summarizes participation, volunteering, and the other substantive measures retained for descriptive and interpretive context.

**Table 1:** Characteristics of the resident analytical sample.

Variable	Category	n	%
**Gender**	Female	1574	58.56
**Gender**	Male	1114	41.44
**Age**	0–14 years	9	0.33
**Age**	15–29 years	310	11.53
**Age**	30–45 years	1130	42.04
**Age**	46–60 years	865	32.18
**Age**	61–75 years	336	12.50
**Age**	≥76 years	38	1.41
**Education**	High school or below	1451	53.98
**Education**	Bachelor’s degree	1190	44.27
**Education**	Master’s degree or above	47	1.75
**Monthly income**	No current income source	250	9.30
**Monthly income**	<3,000 yuan	642	23.88
**Monthly income**	3,000–6,000 yuan	1312	48.81
**Monthly income**	6,000–10,000 yuan	352	13.10
**Monthly income**	>10,000 yuan	132	4.91
**Party membership**	No	1778	66.15
**Party membership**	Yes	910	33.85

**Table 2:** Distribution of participation and other substantive measures.

**Participation frequency**	Never				742	27.60	
**Participation frequency**	Rarely				717	26.67	
**Participation frequency**	Occasionally				614	22.84	
**Participation frequency**	Often				393	14.62	
**Participation frequency**	Always				222	8.26	
**Volunteer status**	No				981	36.50	
**Volunteer status**	Yes				1707	63.50	
**Services known**	2688	10.06	6.36	9	13	0	19
**Information channels known**	2688	3.55	1.89	3	3	1	7
**Service windows known**	2688	2.99	1.21	4	2	0	4
**Governance friction**	2688	0.49	0.83	0	1	0	3

### 3.5 Distributional diagnostics and ceiling effect

Before regression modeling, the Q19 mean score was 4.7466 (SD = 0.5010), with a median of 5.000, Q1 = 4.750, Q3 = 5.000, and IQR = 0.250. The distribution was strongly negatively skewed (−2.655) and highly leptokurtic (excess kurtosis = 9.235). Shapiro-Wilk rejected normality (W = 0.579, p < .001), and histogram and Q-Q diagnostics showed the same concentration near the upper boundary. Across the 20 items, the proportion selecting category 5 ranged from 74.11% to 83.71%. Table 3 summarizes the distributional, reliability, and factor-analytic diagnostics.

**Table 3:** Distributional and measurement properties of the Q19 evaluation scale.

Panel A. Scale-level diagnostics					
Statistic	Value				
**Number of items**	20				
**Mean Q19 score**	4.7466				
**Standard deviation**	0.5010				
**Median**	5.000				
**Q1/ Q3**	4.750/ 5.000				
**Interquartile range**	0.250				
**Skewness**	−2.655				
**Excess kurtosis**	9.235				
**Shapiro-Wilk W**	0.579 (p < .001)				
**Strict top box, n (%)**	1,701 (63.281%)				
**Cronbach’s alpha (95% CI)**	0.986 [0.985, 0.987]				
**Overall KMO**	0.984				
**Bartlett test**	chi-square = 80,067.60, df = 190, p <.001				
**Factors retained**	1				
**Extraction/ rotation**	MINRES/ none				
**Variance explained by Factor 1**	78.64%				
**Scale-factor correlation**	0.9995				
**Panel B. Item-level one-factor results**					
**Item**	**Variable**	**Loading**	**Communality**	**Uniqueness**	**Item KMO**
**Location Access**	Q19_01location_access	0.786	0.618	0.382	0.981
**Building Identity**	Q19_02building_identity	0.833	0.695	0.305	0.979
**Indoor Environment**	Q19_03indoor_environment	0.875	0.765	0.235	0.982
**Red Symbol Visibility**	Q19_04red_symbol_visibility	0.837	0.7	0.3	0.98
**Staff Politeness**	Q19_05staff_politeness	0.883	0.78	0.22	0.983
**Staff Professionalism**	Q19_06staff_professionalism	0.899	0.809	0.191	0.986
**Staff Patience**	Q19_07staff_patience	0.922	0.85	0.15	0.977
**Staff Efficiency**	Q19_08staff_efficiency	0.919	0.845	0.155	0.983
**Staff Clarity**	Q19_09staff_clarity	0.917	0.842	0.158	0.987
**Political Publicity**	Q19_10political_publicity	0.909	0.826	0.174	0.989
**Service Platform Value**	Q19_11service_platform_value	0.916	0.838	0.162	0.989
**Time Energy Saving**	Q19_12time_energy_saving	0.914	0.835	0.165	0.986
**Need Fulfillment**	Q19_13need_fulfillment	0.854	0.729	0.271	0.986
**Digital Service Efficiency**	Q19_14digital_service_efficiency	0.902	0.814	0.186	0.987
**Open Accessibility**	Q19_15open_accessibility	0.891	0.795	0.205	0.992
**Outdoor Activity Supply**	Q19_16outdoor_activity_supply	0.896	0.802	0.198	0.975
**Indoor Activity Supply**	Q19_17indoor_activity_supply	0.909	0.826	0.174	0.975
**Elderly Activity Supply**	Q19_18elderly_activity_supply	0.885	0.783	0.217	0.984
**Children Activity Supply**	Q19_19children_activity_supply	0.877	0.769	0.231	0.986
**Secretary Leadership Satisfaction**	Q19_20secretary_leadership_satisfaction	0.898	0.806	0.194	0.991

One-factor solution estimated by MINRES without rotation. Loadings are reported to three decimals.

The outcome was not transformed solely to obtain normality. The departure from normality was treated as a substantive feature of a bounded, ceiling-concentrated scale. The continuous mean score was retained for interpretability; the factor-score specification assessed measurement robustness; and the binary top-box specification directly modeled the upper-bound mass.

### 3.6 Statistical analysis

Resident observations were clustered within PMSCs. The primary continuous-outcome analysis used ordinary least squares with standard errors clustered at the center level across 140 resident analytical clusters, with finite-sample cluster correction and t-based inference. The center identifier was used only as a clustering variable.

Let i index residents and j index centers. Participation was represented by indicators for rarely, occasionally, often, and always participating, with never participating omitted. The primary models were:


$$\begin{array}{cc} {\text{Y}}_{\text{ij}}= & \beta_{\text{0}}+\beta_{\text{1}}{\text{Rarely}}_{\text{ij}}+\beta_{\text{2}}{\text{Occasionally}}_{\text{ij}}+\beta_{\text{3}}{\text{Often}}_{\text{ij}}+\beta_{\text{4}}{\text{Always}}_{\text{ij}}+\beta_{\text{5}}{\text{Male}}_{\text{ij}}+\text{Age}-\text{category terms}\\ & +\text{Education}-\text{category terms }+\text{Income}-\text{category terms}+\beta_{\text{6}}{\text{PartyMember}}_{\text{ij}}+\epsilon_{\text{ij}} \end{array}$$ (1)

$$\begin{array}{c} {\text{F}}_{\text{ij}}=\beta_{\text{0}}\text{F}+\beta_{\text{1}}\text{F}{\text{Rarely}}_{\text{ij}}+\beta_{\text{2}}\text{F}{\text{Occasionally}}_{\text{ij}}+\beta_{\text{3}}\text{F}{\text{Often}}_{\text{ij}}+\beta_{\text{4}}\text{F}{\text{Always}}_{\text{ij}}+\text{the same demographic covariates}+\epsilon_{\text{ij}}\text{F} \end{array}$$ (2)

$$\begin{array}{c} \text{log}[\pi_{\text{ij}}/(\text{1}-\pi_{\text{ij}})]=\alpha_{\text{0}}+\alpha_{\text{1}}{\text{Rarely}}_{\text{ij}}+\alpha_{\text{2}}{\text{Occasionally}}_{\text{ij}}+\alpha_{\text{3}}{\text{Often}}_{\text{ij}}+\alpha_{\text{4}}{\text{Always}}_{\text{ij}}+\text{ the same demographic covariates} \end{array}$$ (3)

$$\begin{array}{c} {\text{Y}}_{\text{ij}}=\mu +{\text{u}}_{\text{j}}+{\text{e}}_{\text{ij}};\text{ ICC}=\sigma^{\text{2}}\text{u}/\;(\sigma^{\text{2}}\text{u}+\sigma^{\text{2}}\text{e}) \end{array}$$ (4)

In Equation 1, Y is the 1–5 Q19 mean score; in Equation 2, F is the standardized MINRES factor score. Both continuous-outcome models used center-clustered standard errors. The omitted categories were 30–45 years for age, high school or below for education, and 3,000–6,000 yuan for income.

The strict top-box outcome was estimated with a binomial generalized estimating equation (GEE) using a logit link, center grouping, an exchangeable working correlation, and robust sandwich covariance. Exponentiated coefficients are reported as odds ratios. An unconditional random-intercept model was used only to quantify between-center variation. The preferred REML/Powell estimate yielded ICC = 0.011, with a one-way ANOVA method-of-moments estimate of 0.007 as validation.

### 3.7 Sensitivity analyses

Sensitivity analyses examined whether the participation association depended materially on influential observations, small clusters, top-box definition, or the GEE working-correlation assumption. Cook’s-distance analyses excluded the single most influential observation and the ten most influential observations. Center-size restrictions retained centers with at least 2, 5, or 10 respondents. The exchangeable GEE was compared with an independence working correlation, and an alternative near-ceiling definition used Q19 mean ≥ 4.8.

Two deliberately stringent response-pattern stress tests excluded all respondents with all-5 Q19 responses and, separately, all respondents with constant responses across the 20 items. Because these restrictions were directly related to the outcome and removed a large, systematically selected share of respondents, they were treated only as sensitivity analyses, not as replacement samples.

All results are interpreted as adjusted within-sample associations. Center-clustered standard errors, GEE, ICC estimation, and sensitivity analyses address statistical dependence or specification sensitivity; they do not remove self-selection, reverse causality, common-method covariance, omitted-variable endogeneity, or other limitations of the cross-sectional nonprobability design.

## 4. Results

### 4.1 Descriptive statistics and ceiling pattern

The analytical sample comprised 2,688 residents from 140 centers. Participation was distributed across the five categories as follows: 27.60% never, 26.67% rarely, 22.84% occasionally, 14.62% often, and 8.26% always participated. Center-level sample size ranged from 1 to 83 respondents, with 12 singleton centers.

Resident evaluation was concentrated at the upper boundary. The mean Q19 score was 4.7466 (SD = 0.5010), the median was 5.000, and the IQR was 0.250 (Q1 = 4.750; Q3 = 5.000). Shapiro-Wilk rejected normality (W = 0.579, p < .001), and 1,701 respondents (63.281%) rated all 20 items as 5, meeting the strict top-box definition. Descriptive characteristics are reported in Table 1, and distributional diagnostics in Table 3.

### 4.2 Reliability and factor structure

The 20-item evaluation scale showed very high internal consistency (Cronbach’s α = 0.9862, 95% CI [0.985, 0.987]) and strong factorability (KMO = 0.9838; Bartlett’s χ² = 80,067.596, p < .001).

Factor-retention diagnostics supported one dominant factor. The first eigenvalue was 15.937 and the second 0.747; in the 500-iteration parallel analysis, only the first observed eigenvalue exceeded the random 95th percentile. The one-factor MINRES solution produced loadings of 0.786–0.922 and accounted for 78.64% of variance. The standardized factor score correlated 0.9995 with the mean scale score (Table 3).

### 4.3 Main center-clustered OLS model

The main OLS model used the Q19 mean score with center-clustered standard errors across 140 centers. Model R² was 0.1241 (adjusted R² = 0.1186), and participation frequency was jointly associated with evaluation, F(4,139) = 42.47, p < .001. Relative to never participating, adjusted evaluation scores were higher for rarely (β = 0.2975, 95% CI [0.238, 0.357]), occasionally (β = 0.3702 [0.311, 0.430]), often (β = 0.3430 [0.272, 0.414]), and always participating (β = 0.4064 [0.340, 0.473]); all p < .001. The pattern was positive across categories but not perfectly monotonic because the estimate for often participating was slightly below that for occasionally participating.

### 4.4 Factor-score robustness model

Replacing the mean score with the standardized one-factor score produced a similar pattern. Model R² was 0.1233 (adjusted R² = 0.1177), and participation remained jointly associated with the outcome, F(4,139) = 41.83, p < .001. Relative to never participating, estimates were 0.5932 SD units for rarely (95% CI [0.4744, 0.7120]), 0.7377 for occasionally [0.6186, 0.8568], 0.6817 for often [0.5401, 0.8233], and 0.8087 for always participating [0.6754, 0.9419]; all p < .001. Table 4 reports full estimates for the mean-score and factor-score models.

**Table 4:** Adjusted associations with resident-reported evaluation in continuous-outcome models.

Predictor	Mean-score beta	95% CI	p	Factor-score beta	95% CI	p
**Participation: Rarely vs Never**	0.297	[0.238, 0.357]	<.001	0.593	[0.474, 0.712]	<.001
**Participation: Occasionally vs Never**	0.370	[0.311, 0.430]	<.001	0.738	[0.619, 0.857]	<.001
**Participation: Often vs Never**	0.343	[0.272, 0.414]	<.001	0.682	[0.540, 0.823]	<.001
**Participation: Always vs Never**	0.406	[0.340, 0.473]	<.001	0.809	[0.675, 0.942]	<.001
**Male vs Female**	−0.009	[-0.049, 0.032]	.674	−0.018	[-0.098, 0.061]	.648
**Age: 0–14 vs 30–45**	−0.299	[-1.028, 0.430]	.419	−0.596	[-2.063, 0.871]	.423
**Age: 15–29 vs 30–45**	0.068	[0.008, 0.128]	.027	0.134	[0.014, 0.254]	.029
**Age: 46–60 vs 30–45**	−0.092	[-0.156, -0.028]	.005	−0.181	[-0.308, -0.054]	.006
**Age: 61–75 vs 30–45**	−0.001	[-0.082, 0.080]	.982	−0.002	[-0.162, 0.158]	.982
**Age: ≥ 76 vs 30–45**	0.015	[-0.116, 0.145]	.826	0.031	[-0.228, 0.289]	.815
**Bachelor’s vs high school or below**	0.017	[-0.041, 0.074]	.570	0.034	[-0.082, 0.149]	.567
**Master’s+ vs high school or below**	0.079	[-0.077, 0.234]	.320	0.151	[-0.160, 0.462]	.338
**No income vs 3,000–6,000 yuan**	−0.073	[-0.155, 0.008]	.078	−0.147	[-0.312, 0.018]	.080
**<3,000 vs 3,000–6,000 yuan**	−0.021	[-0.073, 0.030]	.412	−0.043	[-0.146, 0.060]	.409
**6,000–10,000 vs 3,000–6,000 yuan**	−0.022	[-0.082, 0.037]	.459	−0.048	[-0.168, 0.072]	.431
**>10,000 vs 3,000–6,000 yuan**	−0.041	[-0.172, 0.090]	.536	−0.081	[-0.341, 0.180]	.542
**Party member vs non-member**	0.011	[-0.030, 0.051]	.603	0.021	[-0.060, 0.103]	.608

N = 2,688; 140 centers. OLS with center-clustered standard errors. Reference categories: never participating, female, age 30–45 years, high school or below, income 3,000–6,000 yuan, and non-Party member. R² (adjusted R²): mean-score = 0.124 (0.119); factor-score = 0.123 (0.118).

### 4.5 Strict top-box binomial GEE

The strict top-box GEE showed an overall association between participation and maximum evaluation, Wald χ²(4) = 145.04, p < .001. Relative to never participating, odds ratios were 1.829 for rarely (95% CI [1.493, 2.242]), 2.722 for occasionally [2.167, 3.419], 3.057 for often [2.310, 4.047], and 5.512 for always participating [3.583, 8.481]; all p < .001. Table 5 reports the full GEE covariate estimates and standardized participation-specific probabilities.

**Table 5:** Strict top-box GEE estimates and standardized adjusted probabilities.

Panel A. GEE odds ratios			
Predictor	OR	95% CI	p
**Participation: Rarely vs Never**	1.829	[1.493, 2.242]	<.001
**Participation: Occasionally vs Never**	2.722	[2.167, 3.419]	<.001
**Participation: Often vs Never**	3.057	[2.310, 4.047]	<.001
**Participation: Always vs Never**	5.512	[3.583, 8.481]	<.001
**Male vs Female**	0.965	[0.805, 1.158]	.703
**Age: 0–14 vs 30–45**	0.688	[0.164, 2.894]	.610
**Age: 15–29 vs 30–45**	1.510	[1.104, 2.064]	.010
**Age: 46–60 vs 30–45**	0.572	[0.462, 0.708]	<.001
**Age: 61–75 vs 30–45**	0.868	[0.640, 1.177]	.361
**Age: ≥ 76 vs 30–45**	0.677	[0.355, 1.293]	.238
**Bachelor’s vs high school or below**	1.229	[0.974, 1.550]	.082
**Master’s+ vs high school or below**	2.474	[1.125, 5.442]	.024
**No income vs 3,000–6,000 yuan**	0.840	[0.618, 1.142]	.266
**<3,000 vs 3,000–6,000 yuan**	0.836	[0.648, 1.079]	.168
**6,000–10,000 vs 3,000–6,000 yuan**	0.906	[0.699, 1.174]	.455
**>10,000 vs 3,000–6,000 yuan**	1.388	[0.892, 2.159]	.146
**Party member vs non-member**	0.979	[0.807, 1.187]	.831
**Panel B. Standardized adjusted probabilities by participation frequency**			
**Participation**	**Adjusted probability, % (95% CI)**	**Difference vs never, pp (95% CI)**	
**Never**	47.82 [44.41, 51.24]	Reference	
**Rarely**	62.03 [58.54, 65.52]	14.20 [9.46, 18.95]	
**Occasionally**	70.53 [66.80, 74.26]	22.71 [17.78, 27.64]	
**Often**	72.81 [68.30, 77.31]	24.98 [19.31, 30.65]	
**Always**	82.59 [77.14, 88.04]	34.77 [27.97, 41.57]	

Binomial GEE with logit link, center grouping (140 centers), exchangeable working correlation, and robust sandwich covariance; N = 2,688. OR = odds ratio; CI = confidence interval; pp = percentage points. Probabilities are standardized adjusted predictions. Overall participation test: Wald χ²(4) = 145.04, p < .001.

Standardized adjusted probabilities were 47.82% for never, 62.03% for rarely, 70.53% for occasionally, 72.81% for often, and 82.59% for always participating. The difference between always and never participating was 34.77 percentage points (95% CI [27.97, 41.57]). Fig 1 displays these adjusted probabilities and their 95% confidence intervals.

**Fig 1:**
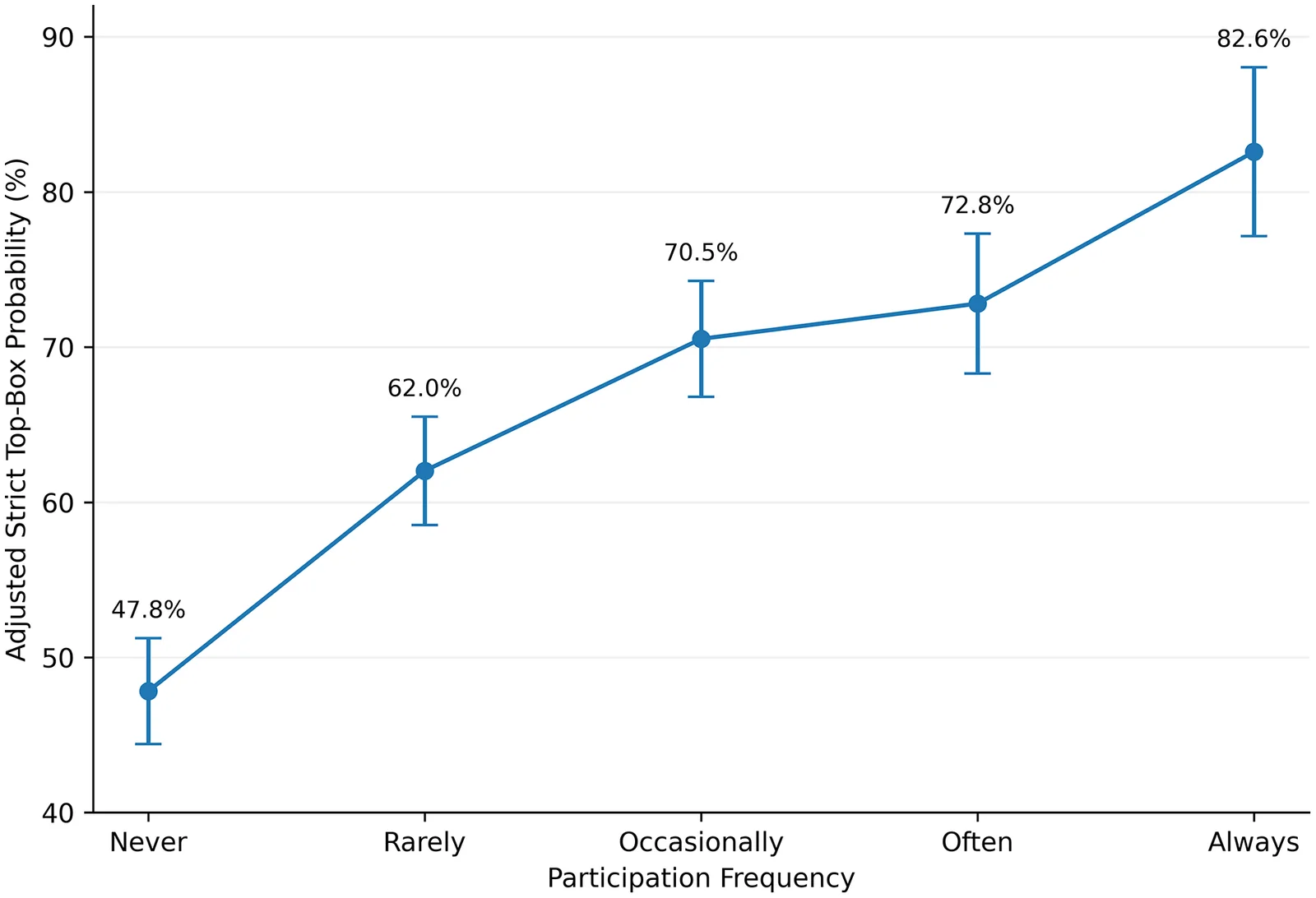
Standardized adjusted probability of a strict top-box evaluation by participation frequency. Points show GEE-based adjusted probabilities; error bars indicate 95% confidence intervals.

### 4.6 ICC and center-level variation

The unconditional random-intercept model estimated center-level variance of 0.002866 and residual variance of 0.248116, yielding ICC = 0.01142 (reported as 0.011). A one-way ANOVA method-of-moments estimate was 0.00728. Thus, between-center variance was small, at about 1% of total variance, but center-aware inference was retained because a small ICC does not justify assuming independence within centers.

### 4.7 Sensitivity analyses

The participation association was stable across influential-observation and center-size restrictions. Excluding the single largest Cook’s-distance observation changed participation coefficients by at most about 0.0032. Excluding the ten most influential observations (N = 2,678) produced coefficients of 0.2809, 0.3517, 0.3322, and 0.3841 for rarely, occasionally, often, and always participating, respectively; all remained positive with p < .001. Restricting the sample to centers with at least 10 respondents retained N = 2,608 across 118 centers and produced coefficients of 0.3034, 0.3778, 0.3528, and 0.4231; less restrictive thresholds yielded the same substantive conclusion.

The top-box result was also stable to alternative specifications. Under an independence working correlation, odds ratios were 1.8388, 2.7213, 3.0523, and 5.4736 (joint Wald χ²(4) = 145.23, p < .001). A broader near-ceiling definition (Q19 mean >= 4.8) retained the same pattern. Extreme response-pattern exclusions likewise preserved direction and statistical significance, although they produced highly selected samples and were treated only as stress tests. Fig 2 summarizes the participation coefficients across the principal OLS sensitivity specifications.

**Fig 2:**
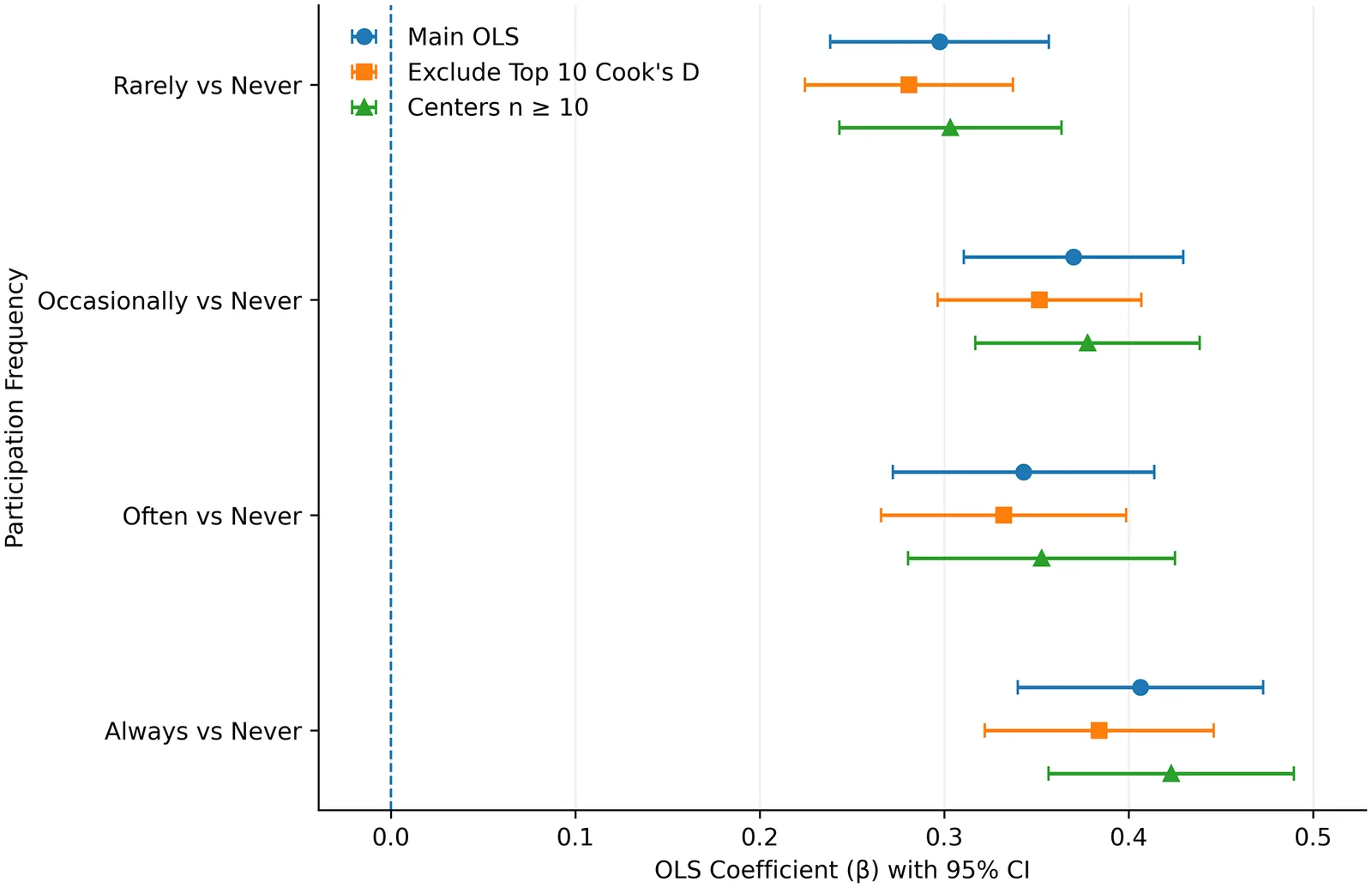
Participation coefficients across principal OLS sensitivity specifications. Points are coefficients relative to never participating; horizontal lines indicate 95% confidence intervals.

## 5. Discussion

### 5.1 Core empirical pattern

The central finding is a positive adjusted association between participation frequency and resident-reported evaluation. Relative to nonparticipants, all four participation categories reported higher mean evaluation scores after demographic adjustment and center-clustered inference. The same broad pattern appeared with the standardized factor score and the strict top-box GEE, and it persisted across the completed sensitivity analyses.

The pattern should not be read as a simple linear dose-response relationship. In the continuous models, the estimate for often participating was slightly lower than that for occasionally participating, although the always category was highest. The evidence therefore supports a robust contrast between participation and nonparticipation, not a claim that each successive participation category produces a fixed increase in evaluation.

### 5.2 Participatory embeddedness and reverse causality

The result is consistent with participatory embeddedness as a marker of a closer resident-institution relationship. Research on local participation in China shows that participation takes heterogeneous forms and that formal participation cannot automatically be equated with substantive influence [12–14]. The present study adds a resident-evaluation pattern to this literature: respondents reporting more frequent participation also reported more favorable evaluations.

The direction of this relationship cannot be established from the cross-sectional survey. Residents with favorable prior evaluations may be more willing to participate; participation may increase familiarity and subsequently coincide with higher evaluations; or both may reflect unobserved characteristics such as civic orientation or prior service needs. Center-clustered standard errors address within-center dependence, not causal direction or endogeneity. The participation-evaluation relationship must therefore remain framed as an adjusted association.

### 5.3 Broader governance context

The broader framework distinguishes participation from several other resident-side dimensions. Service-item awareness captures the breadth of services recognized; information-channel visibility concerns routes through which residents learn about or contact a center; service-window recognition captures the legibility of functional entry points; and governance friction records reported problems in participation or service interaction. These constructs should not be collapsed into a single measure of institutional visibility or quality.

Transparency research supports treating citizen-facing information as an interpretive and use-oriented phenomenon [11], while administrative-burden scholarship highlights learning, compliance, and psychological costs in citizen-state interaction [3,15]. In this study, these dimensions provide conceptual and measurement context rather than independently estimated focal associations. This distinction keeps the discussion aligned with the statistical evidence reported here.

### 5.4 Interpretive boundaries and limitations

First, the outcome exhibits a pronounced ceiling effect: the median is 5.000 and 63.281% of respondents meet the strict top-box definition. The factor-score and top-box analyses show that the participation pattern is not unique to one outcome representation, but they do not eliminate the ceiling or demonstrate the absence of response bias. Reported survey evaluation should also not be equated with a fully observed latent judgment or objective institutional performance, because residents may use different expectations and reference standards.

Second, response context remains a plausible concern. Socially desirable or expressive responding was not directly measured, so the study cannot quantify or attribute the high ratings to social-desirability bias. In addition, participation and evaluation were self-reported in the same survey, leaving open common-method covariance and omitted-variable explanations.

Third, the resident survey used field interception and voluntary online participation. This mixed-mode nonprobability design permits self-selection and does not provide known population inclusion probabilities. Residents who were more familiar with or engaged with PMSCs may have been more likely to participate, and mode-specific measurement equivalence was not directly tested [16]. The results should therefore not be used to estimate population-representative evaluation levels or generalized automatically to other localities or PMSC configurations.

Finally, the design is cross-sectional. Reverse causality remains central to interpretation of the participation association. Center-clustered standard errors, center-grouped GEE, and ICC estimation appropriately address within-center statistical dependence, but they do not solve selection bias, reverse causality, omitted-variable endogeneity, or common-method measurement.

### 5.5 Future research

Future studies could strengthen inference through probability-based recruitment, panel or longitudinal data, independent administrative indicators of center performance, and multi-source measurement that separates predictors from outcomes. Experimental or quasi-experimental designs could further clarify temporal and causal ordering where feasible, while mode-specific validation could distinguish selection from measurement differences in mixed-mode surveys. These approaches define the next steps from robust within-sample association toward stronger population and causal inference.

## 6. Conclusion

This study conceptualizes resident-reported evaluation of PMSCs as a survey-based, reference-dependent judgment rather than a direct audit of institutional performance. Despite a severe ceiling distribution, the 20-item scale showed strong reliability and a clear one-factor structure, allowing complementary continuous, factor-score, and strict top-box analyses.

Across center-clustered OLS, the standardized factor-score model, and center-grouped binomial GEE, residents reporting more frequent participation also reported more favorable evaluations than nonparticipants. The association remained substantively stable across sensitivity analyses, while between-center variation in the evaluation score was small.

The contribution is therefore both substantive and methodological: it brings resident-reported evaluation into the study of grassroots governance while demonstrating how reference-dependent interpretation, ceiling-aware outcome specifications, and center-aware inference can produce a more defensible analysis. The evidence remains associational and non-population-representative, but it clarifies a robust participation-evaluation pattern that future longitudinal and probability-based research can test more strongly.
